# Biologically Inspired Complete Coverage Path Planning Algorithm Based on Q-Learning

**DOI:** 10.3390/s23104647

**Published:** 2023-05-11

**Authors:** Xiangquan Tan, Linhui Han, Hao Gong, Qingwen Wu

**Affiliations:** 1 CAS Key Laboratory of On-Orbit Manufacturing and Integration for Space Optics System, Chinese Academy of Sciences, Changchun Institute of Optics, Fine Mechanics and Physics, Chinese Academy of Sciences, Changchun 130033, China; wuqw@ciomp.ac.cn; 2 Research Center for Materials and Optoelectronics, University of Chinese Academy of Sciences, Beijing 100049, China; hanlinhui20@mails.ucas.ac.cn (L.H.); gonghao21@mails.ucas.ac.cn (H.G.)

**Keywords:** complete coverage path planning, biologically inspired neural network, Q-learning, mobile robot

## Abstract

Complete coverage path planning requires that the mobile robot traverse all reachable positions in the environmental map. Aiming at the problems of local optimal path and high path coverage ratio in the complete coverage path planning of the traditional biologically inspired neural network algorithm, a complete coverage path planning algorithm based on Q-learning is proposed. The global environment information is introduced by the reinforcement learning method in the proposed algorithm. In addition, the Q-learning method is used for path planning at the positions where the accessible path points are changed, which optimizes the path planning strategy of the original algorithm near these obstacles. Simulation results show that the algorithm can automatically generate an orderly path in the environmental map, and achieve 100% coverage with a lower path repetition ratio.

## 1. Introduction

Along with the unceasing progress of science and technology, all kinds of mobile robots have been imagined, and these robots are applied to many fields such as room cleaning [1,2,3], exploration [4], search-and-rescue [5,6], and guard patrol [7]. As one of the most important links with robot technology, path planning has become increasingly important to researchers. Path planning can be divided into two categories: the first is point-to-point path planning [8,9], and the other is complete coverage path planning [10]. Point-to-point path planning needs the robot to plan a path line from the start point to the finish point which does not interfere with the obstacles in the environmental map. Complete coverage path planning needs the robot to plan a path in the environmental map which includes all reachable positions and avoids repeated paths. Traditional complete coverage path planning algorithms are mainly as follows: the stochastic and ergodic algorithm [11], the boustrophedon coverage algorithm [12], the interior screw algorithm [13], and the domain decomposition algorithm [14]. These algorithms are easy to operate and the calculation speed is fast; however, when it comes to practical application, these algorithms have poor efficiency and a high coverage repetition ratio for the planned path.

With the rapid development of artificial intelligence-related technologies, many intelligent algorithms are used for the complete coverage path planning domain [15], such as the genetic algorithm [16], the ant colony algorithm [17], and the biologically inspired neural network algorithm. The biologically inspired neural network algorithm has the following advantages: there is no need to train the model and no need to anticipate environmental information; in addition, it operates with fast computing speed. Therefore, it has been widely studied and applied by many scholars. Yang et al. [18] applied the biologically inspired neural network algorithm to the complete coverage path planning domain for the first time. In their algorithm, the map is shown as a grid map, and each grid is considered as a neuron. The adjacent neurons are connected, and the robot’s mobile path is chosen by calculating the neuronal activity value. Zhu et al. [19] improved the biologically inspired neural network algorithm by presenting the Glasius bionic neural network algorithm (GBNN), which reduced the calculating time of algorithm path planning. Lou et al. [20] presented a complete coverage neural network (CCNN) algorithm, which simplified the calculation process of neural activity. It also combined a modified A* algorithm, which effectively lowered the number of path-turning times, and improved the coverage efficiency. Zhang Zhiyuan et al. [21] brought in the domain neuron status criterion, which increased the biologically inspired path planning efficiency near isolated island obstacles. Qian Jinwei et al. [22] combined the interior screw search and the biologically inspired neural network algorithm, which reduced the calculating time of the path repetition ratio. Curiac et al. [23] used the 2D Arnold cat map as a primary positional entropy source, and proposed a new bio-inspired method to generate deceptive paths for mobile robots to complete patrolling missions under the threat of confrontation. Moysis et al. [24] proposed a simple three-parameter one-dimensional chaotic map, and the proposed pseudo-random bit generator was applied to the problem of chaotic path planning for a UAV exploring a 3D area. Moysis et al. [25] proposed a simple, short, and efficient chaotic path planning algorithm for autonomous mobile robots to cover a given terrain using chaotic and unpredictable motion.

On the other hand, the rapid development of reinforcement learning and deep learning brings a whole new solution to path planning. Ai et al. [26] presented a complete coverage path planning algorithm based on reinforcement learning, which modeled optimized path choice by a reward function with multiclass constraints. Dong Jixin [27] presented a path planning algorithm based on deep learning, which modeled a perceived grid map by coiling base, and he set up an intelligent grid based on resnet-18 grid structure, then accomplished complete coverage on static and dynamic maps by corresponding training. The reinforcement learning method possesses a strong ability for decision making and can find the global optimal solution; however, it costs much more time during the learning process than a generic algorithm, so its application has not been widely used.

Among all aforementioned algorithms, the complete coverage path planning algorithm has the fastest calculating speed, but the path planning repetition ratio is high in complex environments; intelligent algorithms considering environmental information can plan orderly paths, but have the problem of long calculating times. In response to the issue and research contents, this paper presents a biologically inspired neural network algorithm based on Q-learning, which proceeds with path planning based on the biologically inspired neural network algorithm, and when it comes to the position change of a reachable path point, processes path optimization by adopting Q-learning to avoid falling into a locally optimal solution, thus reducing the path repetition ratio, while still ensuring complete coverage, achieving the desired path planning results. The rest of this paper is organized as follows. In Section 2, the complete coverage path planning algorithm based on a biologically inspired neural network is described in detail. In Section 3, the improved algorithm based on Q-learning is introduced. Simulation results and discussions in different environmental maps are presented in Section 4. Finally, the conclusion is given in Section 5.

## 2. Biologically Inspired Neural Network Algorithm

The complete coverage path planning issue can be described as follows: on an environmental map, the mobile robot sets off at the starting point, avoids obstacles, passes by all the reachable position points in an orderly way on the environmental map, and reduces path repetition as much as possible. The biologically inspired neural network algorithm has many advantages such as the following: simple calculation, no need for model training, and the mobile robot can plan a 100% coverage ordered path under the restricted condition of no motion template or energy loss function; therefore, it is widely used in the indoor robot complete coverage path planning domain.

### 2.1. Biologically Inspired Neural Network Model

Physicists Hodgkin and Huxley developed the famous biological nerve cell membrane circuit model [28] to explain the signal transmission issue in nervous systems, where the dynamic membrane voltage $V_{m}$ can be expressed as the following formula:

(1) $$C_{m}\frac{dV_{m}}{dt}=-\left(E_{p}+V_{m}\right)g_{p}+\left(E_{Na}-V_{m}\right)g_{Na}-\left(E_{k}+V_{m}\right)g_{K}$$

where *C_m_* is the membrane capacitance, *E_k_*, *E_na_*, *E_p_*, *g_k_*, *g_na_*, and *g_p_* are electric potentials and the electric conductance of potassium ions, sodion, and the passive leakage current in cell membranes, respectively. Grossberg made a summing based on Equation (1), and set up the neural dynamic network model, and created the famous shunting equation:

(2) $$\frac{dx_{i}}{dt}=-Ax_{i}+\left(B-x_{i}\right)S_{i}^{e}(t)-\left(D+x_{i}\right)S_{i}^{i}(t)$$

where $x_{i}$ can be considered as the neural activity value of the neuron i; *A*, *B*, and *D* are the attenuation ratio, the upper bound, and the lower bound of the neural activity value, respectively; $S_{i}^{e}$ and $S_{i}^{i}$ are the external stimulus and the inhibiting input of the neuron, respectively. The fractional flow Equation (2) is widely used in the domain of visual perception and motion control.

**Figure 1:**
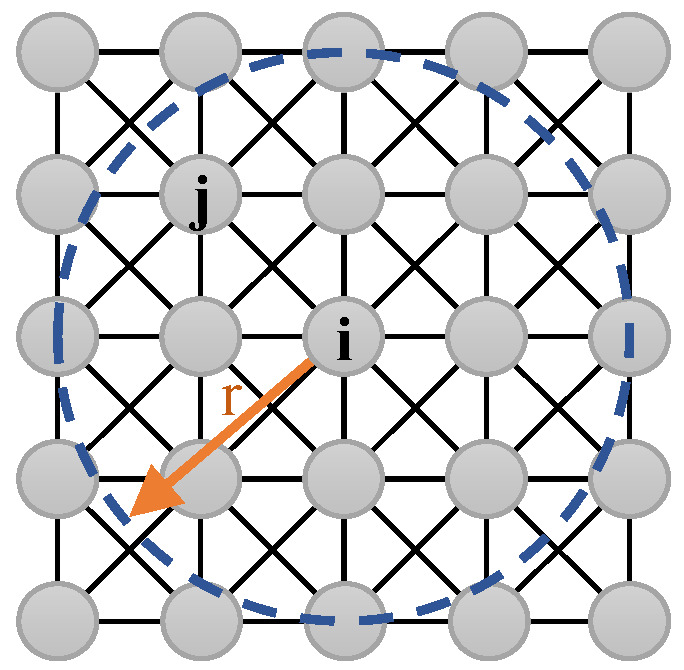
The neural network’s model.

Yang et al. [29] set up a biologically inspired neural network based on Equation (2), and applied it to the path planning domain, which achieved good results. In a neural network, the size of the neuron activity value is decided by the following formula:

(3) $$\begin{array}{c} \frac{dx_{i}}{dt}=-A_{i} \end{array}+\left(B-x_{i}\right)\left({\left[I_{i}\right]}^{+}+\overset{k}{\underset{j=1}{\sum }}\omega_{ij}{\left[x_{j}\right]}^{+}\right)-\left(D+x_{i}\right){\left[I_{i}\right]}^{-}$$

where $x_{i}$ and $x_{j}$ are the neuron activity values of neurons i and j, k is the amount of all the adjacent neurons of neuron i, and the representative content of A, B, and D are the same as in Equation (2). ${\left[I_{i}\right]}^{+}+\overset{k}{\underset{j=1}{\sum }}\omega_{ij}{\left[x_{j}\right]}^{+}$ and ${\left[I_{i}\right]}^{-}$ are the external stimulus and the inhibiting input of the neuron. The functions ${\left[f\right]}^{+}$ and ${\left[f\right]}^{-}$ are defined as ${\left[f\right]}^{+}=\max\left\{f,0\right\}$ and ${\left[f\right]}^{-}=\max\left\{-f,0\right\}$, and $\omega_{ij}$ is the weight connection of neurons i and j, which can be defined as follows:

(4) $$\omega_{ij}=\left\{\begin{array}{cc} \frac{\mu }{\left|i-j\right|} & 0<|i-j|<r\\ 0 & |i-j|\geq r \end{array}\right.$$

where $\left|i-j\right|$ is the Descartes distance of the neurons on the grid map, and μ is a positive constant. The value of r decides the connection range of the neurons, as shown in Figure 1, when the value of r=2, and the neuron i connects to 8 neurons around i. The external input $I_{i}$ in Equation (3) can be defined as follows:

(5) $$I_{i}=\left\{\begin{array}{c} \begin{array}{cc} E & \mathrm{target}\;\mathrm{point}\;\mathrm{of}\;\mathrm{neuron} \end{array}\\ \begin{array}{cc} -E & \mathrm{obstacle}\;\mathrm{point}\;\mathrm{of}\;\mathrm{neuron} \end{array}\\ \begin{array}{cc} 0 & \mathrm{other}\;\mathrm{point}\;\mathrm{of}\;\mathrm{neuron} \end{array} \end{array}\right.$$

where E is a large number positive constant, to express a large external input.

The above Equation (3) can ensure that the positive neuronal activity can be spread into the entire neural network by the connection of neurons, and the negative neuronal activity can be limited to the nearby obstacles to block signal transmission. Therefore, using the fractional flow formula to define the model, the mobile robot can automatically choose the next moment’s moving direction by the neuron activity value of the neural network, then complete the path planning. The above neural network model can be described in Figure 1.

### 2.2. Biologically Inspired Complete Coverage Path Planning

Yang et al. [8] proceeded with path point optimization selection on the above biologically inspired neural network model, and applied it to the complete coverage path planning domain. In order to generate an ordered and low repetition ratio path, the deciding path points can be defined as the following equation:

(6) $$P_{n}\;⇐\;x_{p_{n}}=\max\left\{x_{j}+cy_{j}\right\},\;j=1,2,\cdots ,k$$

where $P_{c}$ is the current position of the mobile robot, $P_{n}$ is the next moment’s position of the mobile robot, c is a positive constant, $y_{j}$ is the function to define the steering angle of the mobile robot, which can be determined by the following Equation:

(7) $$y_{j}=1-\frac{\Delta \theta_{j}}{\pi }$$

(8) $$\Delta \theta_{j}=\left\{\begin{array}{cc} \Delta \theta & x\leq \pi \\ \left|\Delta \theta_{r}-2\pi \right| & x>\pi \end{array}\right.$$

(9) $$\Delta \theta_{r}=\left|\theta_{j}-\theta_{c}\right|=\left|atan2\left(y_{p_{j}}-y_{p_{c}},x_{p_{j}}-x_{p_{c}}\right)-\right.\left.atan2\left(y_{p_{c}}-y_{p_{p}},x_{p_{c}}-x_{p_{p}}\right)\right|$$

where $P_{p}$ is the next moment’s position of the mobile robot; $\Delta \theta_{j}\in \left[0,\pi \right]$ is the angle variation of the next moment’s moving direction of the mobile robot; $atan2\left(y_{a},x_{a}\right)\in \left(-\pi ,\pi \right]$ is the four quadrant arc-tangent functions to describe the angle variation of the moving direction.

According to the above path point selection method, the biologically inspired complete coverage path planning process is as follows:

(a) Build a neural network model correlated with environmental information, and set up external excitation and inhibiting input according to obstacles in the map and the positions of reachable points;
(b) Iteratively calculate each neuron’s activity value according to the neural network model and external input;
(c) The mobile robot selects the next moment’s path point according to the activity value of the neuron adjacent to the current position and steering angle variation;
(d) When the robot falls into a dead zone, which means that around its current position are obstacles and the path point has been passed, the robot calculates the activity value of the neuron grid adjacent to the current position; if the values are all less than the value of the current position, the robot remains still until there is one path point’s activity value greater than the current position value; then the robot moves into the position of the next moment’s path point;
(e) The mobile robot considers the next moment’s path point as the current position, then estimates if it will accomplish the complete coverage of the environmental map. If the answer is yes, then it is the end of the algorithm; if the answer is no, then go back to step (b).

**Figure 2:**
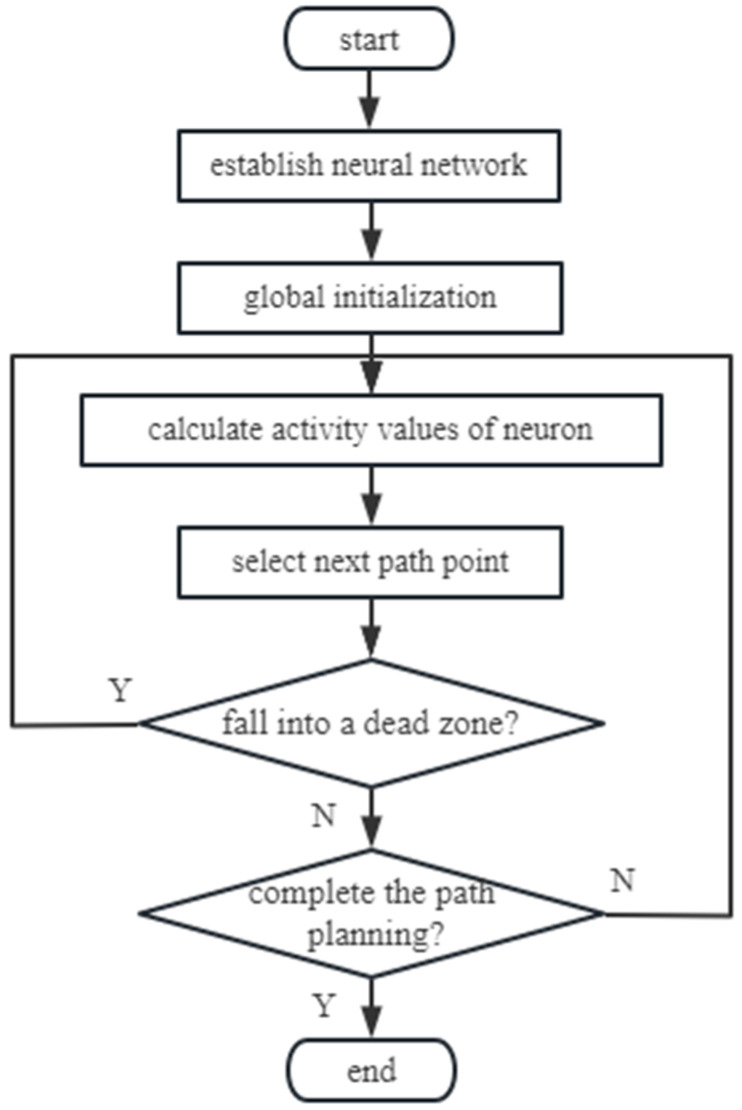
Flow chart of biologically inspired complete coverage path planning algorithm.

According to the above content, a flow chart of the biologically inspired complete coverage path planning algorithm based on Q-learning is shown as follows (Figure 2):

## 3. Improved Algorithm Based on Q-Learning

The biologically inspired neural network algorithm has the merit of simple calculation; however, the repetition ratio of planned paths is very high in an environment with obstacles distributed in complexity; therefore, this paper presents the improved algorithm based on Q-learning, which optimizes the path combined with the environmental map.

### 3.1. Issue Description

**Figure 3:**
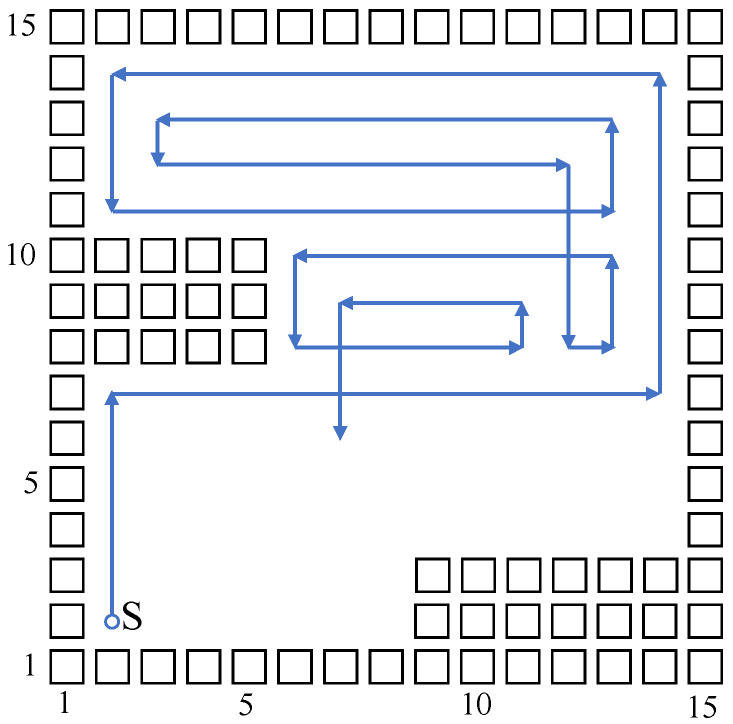
Part of the path planned by the biologically inspired neural network algorithm.

According to the above algorithm principle, build a 15×15 grid map corresponding to the neural network model. The mobile robot starts to plan a path at the starting point (2,2); in order to generate a more ordered path, the neuron of the neural network model only connects to four neurons whose directions are up, down, left, and right, which means the mobile robot can only move towards four directions. Parts of the planned path figure generated by the algorithm are shown in Figure 3, where the black rectangle represents obstacles, and the blue directed line segment represents the planned path.

As shown in Figure 3, the above biologically inspired neural network complete coverage algorithm can avoid obstacles, and moves automatically to uncovered position points; however, the path selection strategy is decided by Equation (6), and therefore, in most cases, the path selected by the robot is only extended in the direction of the last moment, until it meets obstacles or a planned path. The above moving strategy does not consider feature information of the environmental map, and it cannot adjust direction automatically according to the position of obstacles, so the generated path is unordered and the repetition ratio is high; when it comes to a more complex environment, this situation is more distinct. For that, this paper brings in Q-learning to optimize the selection on path direction, to reduce the repetition ratio of the algorithm model.

### 3.2. Principles of the Q-Learning Algorithm

Q-learning is a classic no model reinforcement learning algorithm, which is based on the theory of Markovian decision processes. Markovian decision processes can be defined by a tetrad $\langle S,A,P,R\rangle$, where S represents discrete-time state space, A represents discrete-time motion space, P represents the probability function of state transition, R is the reward function, which represents the reward of state transition after the intellectual body takes an action. The target of the Markovian decision is to find an appropriate strategy, to maximize the cumulative mathematical expectation of the reward function. According to Behrman’s optimal criterion, the optimal index of the optimal strategy is as follows:

(10) $$V^{*}\left(x\right)=\underset{a\in A}{\max}\left[R\left(s,a\right)+\gamma \underset{s^{\prime }\in S}{\sum }P_{s,a}\left(s^{\prime }\right)V\left(s^{\prime }\right)\right]$$

There is a state action function, *Q* function, in Q-learning for evaluation. The function value of $Q\left(s,a\right)$ is the maximum cumulative reward reduced value after the motion a is executed from state S. The optimum strategy of this moment can be defined by the *Q* function, and there is no need to consider the state transition probability P, so by selecting the motion which maximizes the *Q* function from all the motions a in the state S, then the globally optimal strategy π can be obtained. The *Q* function iterative formula based on Behrman criterion is as follows:

(11) $$Q\left(s_{t},a\right)=Q\left(s_{t},a\right)+\alpha \left[r_{t}+\gamma maxQ\left(s_{t+1},a\right)-\right.\left.Q\left(s_{t},a\right)\right]$$

where $\alpha \in \left(0,1\right)$ is the learning rate, $r_{t}$ is the rewards the intelligent body receives after performing the action, and $\gamma \in \left(0,1\right)$ is the discount factor.

**Figure 4:**
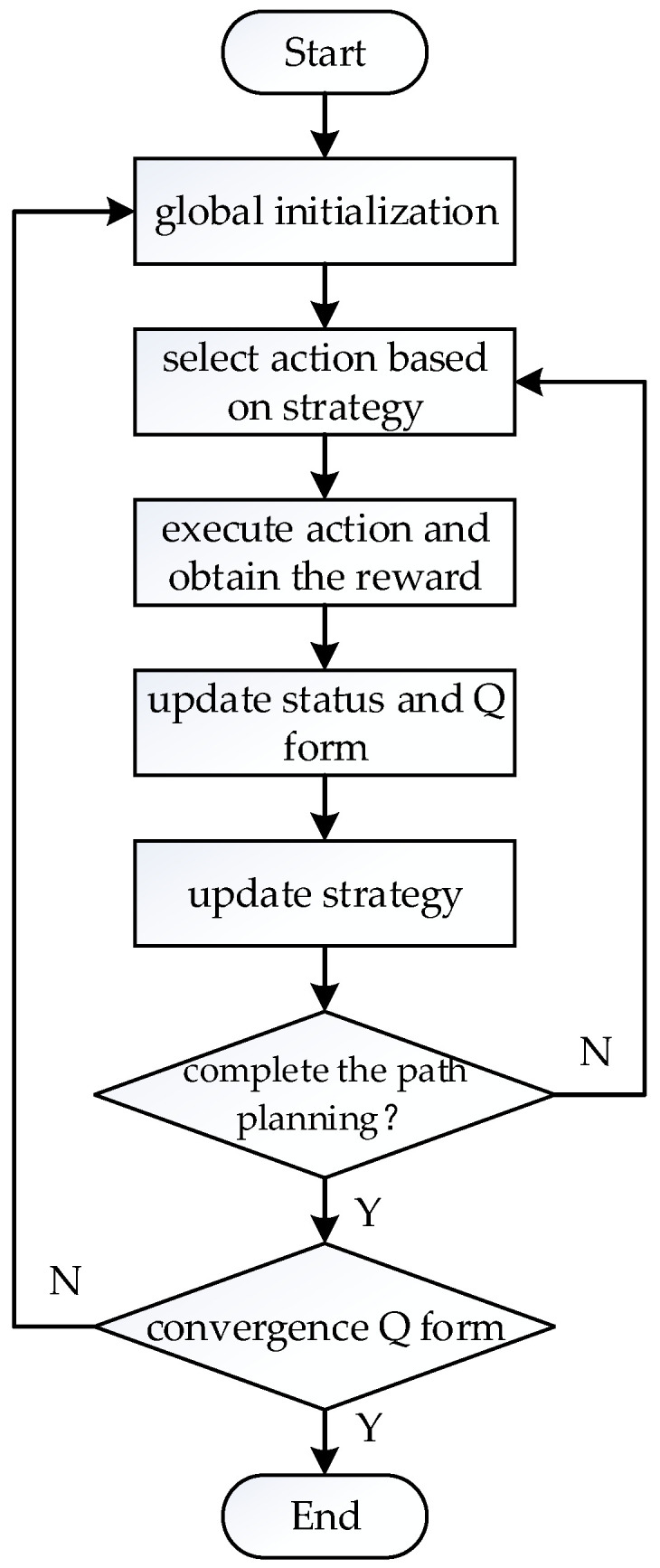
Flow chart of the Q-learning algorithm.

During the learning process, Q-learning sets up a *Q* form whose purpose is to store the *Q* value that corresponds to all the state actions of the intelligent body. The common action selection strategy of Q-learning is the ε− greedy strategy. By setting the greedy factor $\varepsilon \in \left[0,1\right]$, the intelligent body will select the optimal action based on the *Q* form when the motion selection has ε probability, and will select a random action when the motion selection has 1−ε probability, which then gives the intelligent body the ability to explore actively. Through repeated learning, the *Q* form comes to convergence, and then the intelligent body can obtain each state’s optimal action through the *Q* form, which is the output of the optimal policy. The algorithm flow chart of Q-learning is shown in Figure 4.

### 3.3. Algorithm Improvements

**Figure 5:**
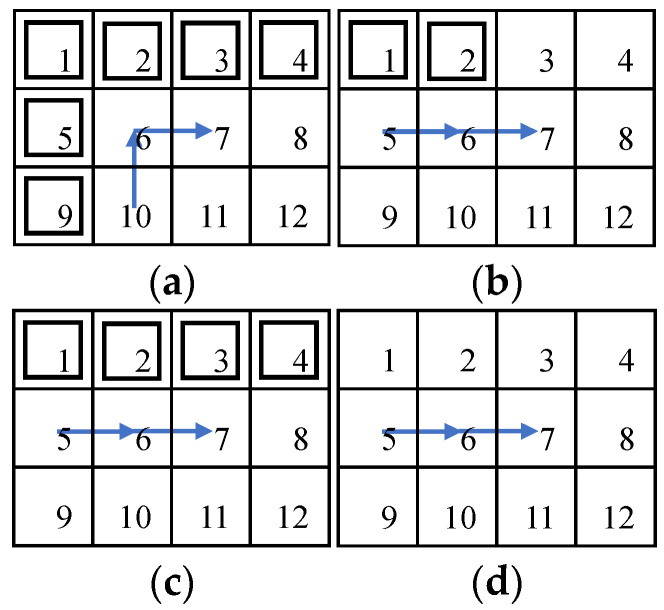
Variations of the reachable positions of the mobile robot in different situations. (**a**) Obstacles on left and top; (**b**) obstacles on top left; (**c**) obstacles on top; (**d**) no obstacles.

Reinforcement learning has a strong ability for learning and decision making; however, applying a reinforcement learning algorithm on complete coverage path planning will create a problem: the convergence speed of the function *Q* is very slow, and sometimes it does not converge due to big environmental maps and sparse learning rewards, so entirely applying the Q-learning algorithm to a global map will need to be improved. The efficiency of the original biologically inspired neural network algorithm is high; however, it does not consider map features near obstacles or around the planned path, so applying the Q-learning algorithm on the area near obstacles and proceeding with strategy optimization can improve efficiency greatly. In order to increase the ratio of convergence of Q-learning, only apply Q-learning when the mobile robot detects that the reachable position points are increasing, and apply the biologically inspired neural network algorithm on the other areas where there is no change in the reachable position points, which can plan the path rapidly and orderly, as shown in Figure 5.

In Figure 5a, the robot in position 6 has only one reachable point, 7, and when the robot reaches position 7, the reachable position points increase to two points, 8 and 11, so this is the moment to apply Q-learning to the path planning. In Figure 5b, the robot in position 6 has two reachable points, 7 and 10, and when the robot reaches position 7, the reachable position points increase to three points, 3, 8, and 11, so this is the moment to apply Q-learning to the path planning. In contrast, in Figure 5c,d, during the mobile robot’s motor process, the number of reachable position points does not increase, so this is the moment to apply the biologically inspired neural network to the path planning. Figure 5 shows the situations that might happen during path planning, and the situations are judged by the number of reachable position points; if the number is increased, then apply Q-learning to the path planning.

In order to reduce algorithm complexity and generate more ordered paths, make a mobile robot that can only move towards up, down, left, and right (four directions) for reinforcement learning and the biologically inspired neural network algorithm. In order to accelerate the *Q* form convergence of this algorithm, the mobile robot is encouraged to change moving direction according to the position of obstacles when the reachable position points increase. The setting of the greedy strategy of the Q-learning process is shown as follows, where $\varepsilon \in \left[0,1\right],\sigma \in \left[0,1\right]$:

(12) $$\pi \left(s_{t}\right)=\left\{\begin{array}{l} argmax\;Q\left(s_{t},a\right)\;\;\;\sigma \leq \varepsilon \\ a\in A\;\;\;\varepsilon <\sigma \leq \varepsilon +2\times \frac{1-\varepsilon }{3}\\ a\in B\;\;\;\varepsilon +\frac{1-\varepsilon }{3}<\sigma \end{array}\right.$$

When the mobile robot chooses a moving direction, determine an exploring factor ε. The robot selects an optimal action in *Q* form based on the probability of the exploring factor ε. Select the action A with a different moving direction from the last moment’s action based on the probability of $2\times \frac{1-\varepsilon }{3}$ and randomly select the action B based on the probability of $\frac{1-\varepsilon }{3}$. At the beginning of learning, the Q form has not come to convergence, and the algorithm tends to explore the environment, which means selecting a smaller ε. After a period of learning, the algorithm tends to make use of the environment, and the number of ε should be set for a large number, to ensure finding the global optimal solution.

**Figure 6:**
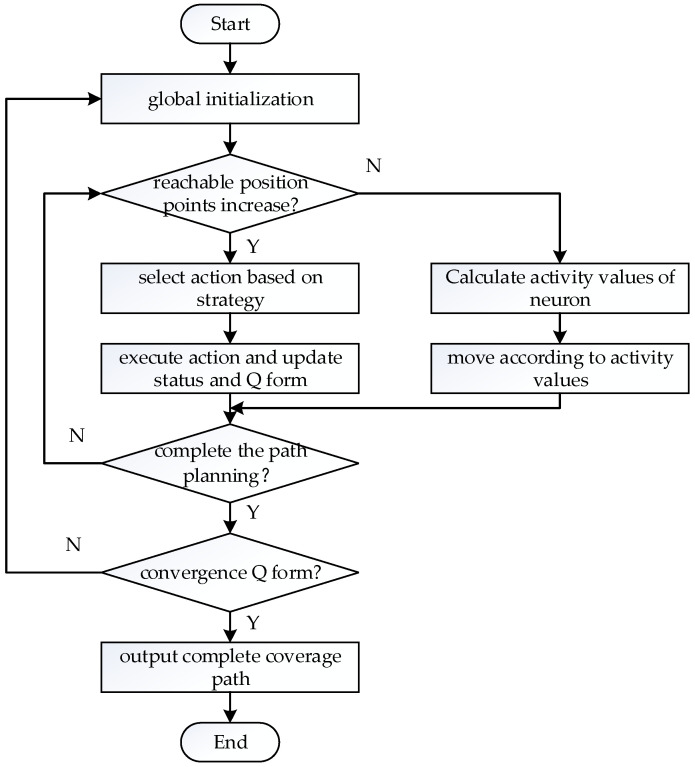
Flow chart of the biologically inspired complete coverage path planning algorithm based on Q-learning.

According to the above content, a flow chart of the biologically inspired complete coverage path planning algorithm based on Q-learning is shown as follows (Figure 6):

Specific steps of the algorithm according to the above algorithm flow are as follows:

(a) Build an environmental map, and then initialize the parameters of the biologically inspired neural network and Q-learning;
(b) Estimate if the reachable position points number of the mobile robot is increasing; if it is not increasing, proceed to step (c); if it is increasing, proceed to step (d).
(c) Calculate each neuron’s activity value of the neural network according to the obstacle distribution in the obstacle environmental map and the planned path based on Equation (3), and then the robot moves according to Equation (6); proceed to step (e);
(d) Select the next mobile strategy according to Q-learning’s greedy algorithm, proceed to moving and update Q form, and proceed to step (e);
(e) Estimate if complete coverage path planning will be accomplished or not; if not, go back to step (b), and continue to plan paths; if yes, proceed to step (f);
(f) Estimate if the Q form has been converged or not; if not, go back to step (a) and proceed with the next learning; if yes, then complete the path planning task, and output the path.

## 4. Simulation Experiment Analysis

In order to verify the feasibility and effectiveness of the biologically inspired complete coverage path planning algorithm based on Q-learning, proceed to the simulation experiment of mobile robot path planning on an indoor environmental map.

### 4.1. Setting of Simulation Conditions

Build a grid map according to the size of the environmental map and obstacle distribution. Set the starting point of the mobile robot. If the robot reaches all the coverage grid points, it is considered as having accomplished the complete coverage path planning.

Set partial parameters of the biologically inspired neural network as follows: in Equation (3), A=100, B=1, D=1, μ=0.8, r=1, output E=100, and in Equation (6), weighting c=0.1. Set partial parameters of Q-learning as follows: set four basic motions in the mobile robot motion space which are up, down, left, and right; and in Equation (11), α=0.5, γ=0.7. The settings of the reinforcement learning process reward are as follows: when the intelligent agent reaches an uncovered position point, the reward value is +3; when the intelligent agent reaches a covered position point, the reward value is −2; when the intelligent agent hits obstacles, the reward value is −10, and the planning task is considered a failure; when the intelligent agent’s moving steps are greater than two times the total grid number, the reward value is −15, and the planning task is considered a failure; when the intelligent agent reaches all the coverage position points, the reward value is +15, and the planning task is considered successful.

### 4.2. Indoor Map Simulation Experiment

**Figure 7:**
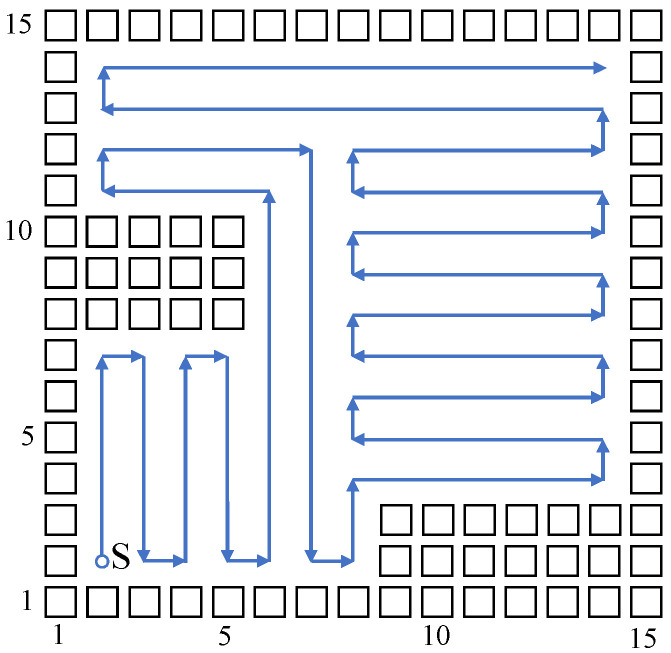
The complete coverage path of the proposed algorithm in the 15×15 grid map.

**Figure 8:**
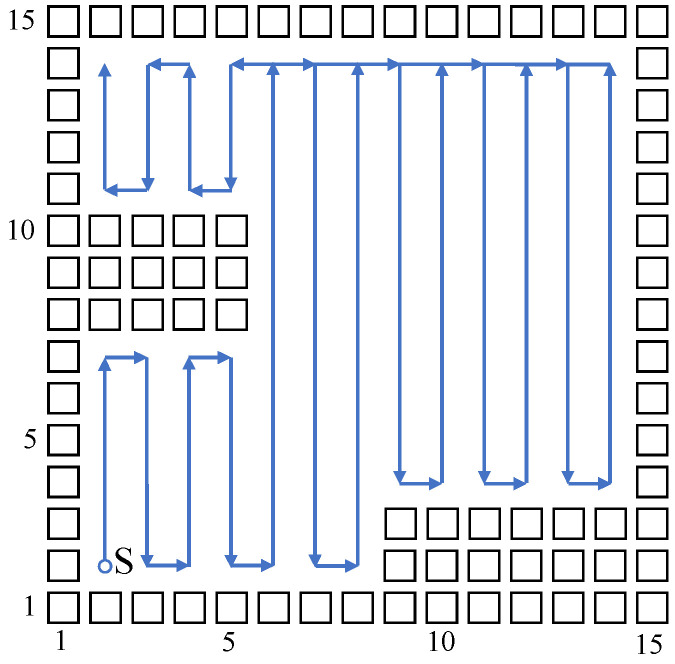
The complete coverage path of the boustrophedon algorithm in the 15×15 grid map.

**Figure 9:**
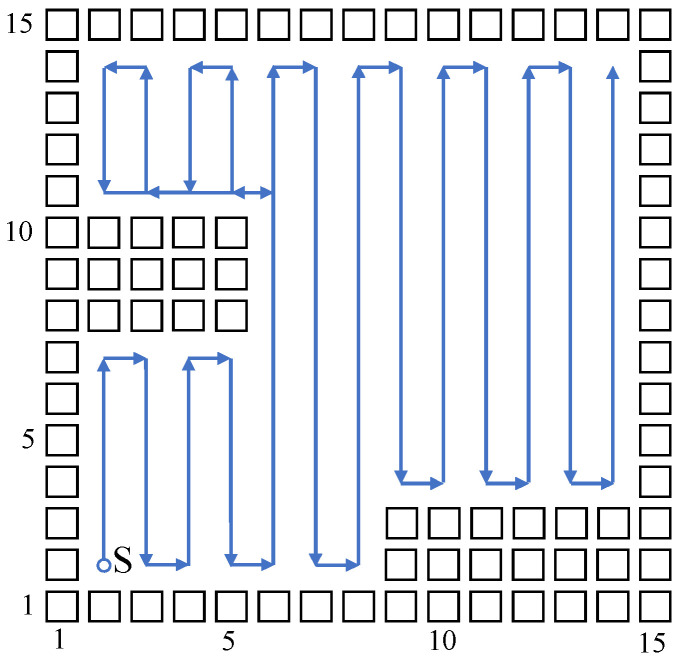
The complete coverage path of the secondary area division algorithm in the 15×15 grid map.

Build a size 15 × 15 grid map according to an indoor environment, shown in Figure 7. The obstacle position information in the grid map is known, shown using a black rectangle. The mobile robot sets off at the starting point (2,2). The planned path is shown using the blue directed line segment. When there are no changes at the mobile robot’s reachable position point, the robot plans a path and moves using the biologically inspired neural network, and the mobile robot sheers off and avoids obstacles according to the neuron activity value corresponding to the grid point. When the reachable position point changes, adopt the Q-learning algorithm to choose a more reasonable path point, and sheer off automatically based on the obstacle map information, and the repetition ratio of the complete coverage path planning is reduced. On the same condition of the same experiment map and starting point, this paper compares the biologically inspired complete coverage path planning algorithm based on Q-learning with the traditional boustrophedon coverage algorithm [4] as shown in Figure 8 and the secondary region partitioning algorithm based on cellular automaton theory [30] as shown in Figure 9; the repetition ratio of path planning, the coverage ratio of path planning, and the number of sheering off times are shown in Table 1. According to the comparison results, on the same conditions, the algorithm proposed in this paper can achieve a 100% coverage ratio, and change path directions automatically based on the position of obstacles; although the sheering times are increased, the path repetition ratio is reduced effectively.

**Figure 10:**
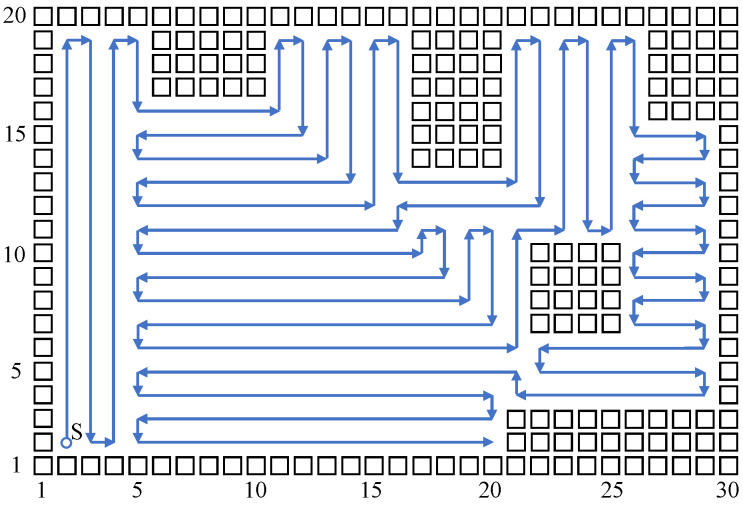
The complete coverage path of the proposed algorithm in the 30×20 grid map.

**Figure 11:**
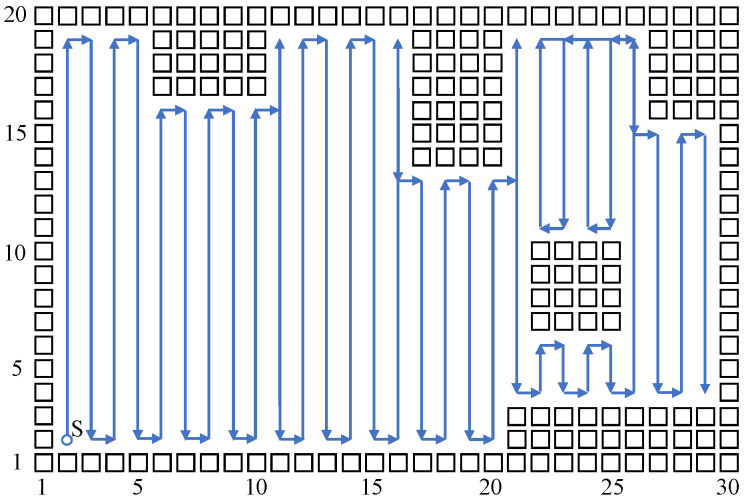
The complete coverage path of the boustrophedon algorithm in the 30×20 grid map.

**Figure 12:**
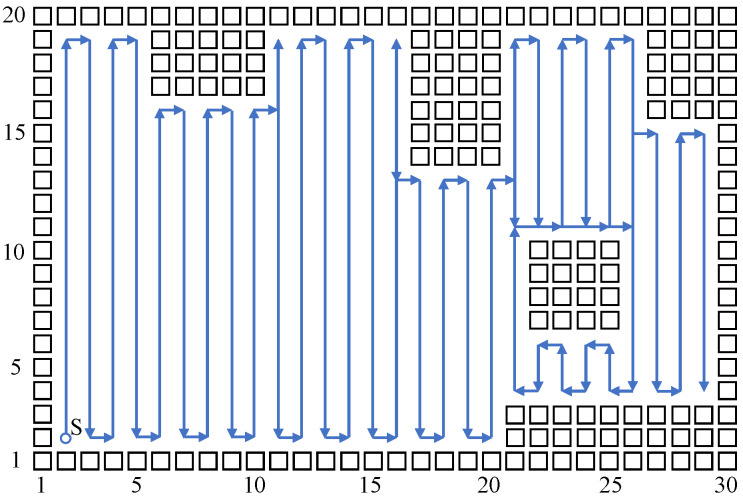
The complete coverage path of the secondary area division algorithm in the 30×20 grid map.

The above simulation results show that the biologically inspired complete coverage path planning algorithm based on Q-learning is very effective, and it can plan a better path compared with the traditional boustrophedon coverage algorithm and the secondary region partitioning algorithm. Furthermore, this paper conducts a simulation experiment on a bigger size indoor environmental map, shown in Figure 10. The sizes of the grid are 30×20, and there are more types of obstacles in the environmental map, such as obstacles near the edge of the map and island-style obstacles. The simulation parameters are set to be the same as in the above simulation process, and the mobile robot starts at the position point (2,2); comparisons with the traditional boustrophedon coverage algorithm as shown in Figure 11 and the secondary region partitioning algorithm as shown in Figure 12 were conducted, and the results are shown in Table 2. The algorithm proposed in this paper can plan a complete coverage path equally with a lower repetition ratio on a bigger size indoor environmental map.

The simulation results show that the improved algorithm is different from the traditional complete coverage path planning algorithms, which show that the improved algorithm proposed in this paper can change planned path direction actively according to the distribution of obstacles on the map. Although the path generated by the improved algorithm has more turns, the path repetition rate is lower, which reduces the energy loss of the mobile robot at work. The improved algorithm has the characteristics of the high computational efficiency of the biologically inspired neural network, and the Q-learning algorithm is introduced to optimize the turning strategy of the path near the obstacles, so that the planned path is more orderly.

## 5. Conclusions

This paper presents a biologically inspired complete coverage path planning algorithm based on Q-learning, which uses Q-learning in areas near obstacles to improve the algorithm of path planning strategy based on the original biologically inspired neural network algorithm. The improved algorithm solves two problems: one is the complex paths and high repetition coverage ratio of the biologically inspired neural network algorithm; the other is avoiding the low computational efficiency of using only Q-learning on complete coverage path planning. The results indicate that the improved algorithm can generate a complete coverage path in an indoor environment without a motion template or energy loss function, and while reducing the path repetition ratio effectively.

This paper conducts research on robot complete coverage path planning in a simpler indoor environment; however, in actual applications, the environment is more complex in general. The Q-learning algorithm tends to find the globally optimal path, so the Q form converges very slowly if there are no constraints. Therefore, there are many involved possibilities during the complete coverage process, and the earlier reinforcement learning exploration stage costs much time, so the calculation speed is very slow and convergence is very difficult in complex and large-scale scenarios. Therefore, the next research content is to expand and improve the algorithm. For example, adding a number of scene restrictions on Q-learning, and optimizing the Q form initial value according to the data on the neuron activity value, may allow this algorithm to achieve complete coverage path planning under the conditions of complex environments such as multi-obstacle scenarios and large-scale scenarios.

## Figures and Tables

**Table 1:** Comparison of complete coverage path data in the 15×15 grid map.

Algorithm	Coverage Ratio (%)	Repetition Ratio (%)	Sheering Times
Improved algorithm in this paper	100	0.00	35
Boustrophedon coverage algorithm	100	5.52	32
Secondary region partitioning algorithm	100	2.76	34

**Table 2:** Comparison of complete coverage path data in the 30×20 grid map.

Algorithm	Coverage Ratio (%)	Repetition Ratio (%)	Sheering Times
Improved algorithm in this paper	100	0.00	83
Boustrophedon coverage algorithm	100	6.21	71
Secondary region partitioning algorithm	100	5.25	72

## Data Availability

All relevant data are within the paper.
